# Prolonged exercise testing in two children with a mild Multiple Acyl-CoA-Dehydrogenase deficiency

**DOI:** 10.1186/1743-7075-2-12

**Published:** 2005-05-20

**Authors:** T Takken, J WH Custers, G Visser, L Dorland, PJM Helders, TJ de Koning

**Affiliations:** 1 Department of Pediatric Physical Therapy & Exercise Physiology, University Hospital for Children and Youth 'Het Wilhelmina Kinderziekenhuis', University Medical Centre Utrecht, Utrecht, The Netherlands; 2 Department of Metabolic Diseases, University Hospital for Children and Youth 'Het Wilhelmina Kinderziekenhuis', University Medical Centre Utrecht, Utrecht, The Netherlands

## Abstract

**Background:**

Multiple Acyl-CoA-Dehydrogenase deficiency (MADD) is an inherited metabolic disorder characterized by impaired oxidation of fatty acids and some amino acids.

**Methods:**

We were interested whether children with MADD could tolerate a prolonged low-intensity exercise test and if this test could have any additional diagnostic value. Therefore, we performed a maximal exercise test and a low-intensity prolonged exercise test in 2 patients with MADD and in 5 control subjects. During a prolonged exercise test the subjects exercised on a cycle ergometer at a constant workload of 30% of their maximum for 90 minutes and heart rate, oxygen uptake, fuel utilization and changes in relevant blood and urinary parameters were monitored.

**Results:**

The tests were tolerated well. During the prolonged exercise test the fatty acid oxidation (FAO) was quite low compared to 5 control subjects, while characteristic metabolites of MADD appeared in plasma and urine.

**Conclusion:**

We suggest that the prolonged exercise test could be of diagnostic importance and might replace the fasting test as a diagnostic procedure in some cases, particularly in patients with anamnestic signs of intolerance for prolonged exercise.

## Introduction

Multiple Acyl-CoA-Dehydrogenase deficiency or Glutaric Acidemia Type II (MADD; McKusick # 231680) is an inborn error due to a deficiency of electron transfer flavoprotein (ETF) or of ETF-ubiquinone oxidoreductase, and is characterized by impaired oxidation of fatty acids (FAO) and some amino acids. Severely affected patients present in the newborn period with multiple congenital abnormalities in association with hypotonia, hepatomegaly, hypoglycemia and severe metabolic acidosis [1]. Patients with milder forms of the disorder present with a variety of symptoms such as hypoglycemia, Reye-like episodes, intermittent vomiting, acidosis and myopathy [1]. In adults, MADD can manifest itself as a lipid storage myopathy [1]. In patients with metabolic diseases, such as glycogen storage diseases [2-4], and FAO disorders such as carnitine palmitoyltransferase II deficiency and very long-chain acyl-coA-dehydrogenase deficiency [5,6] prolonged exercise tests are increasingly getting more attention. However, data in patients with FAO disorders are still scarce. This is the first report investigating fuel utilization and exercise capacity in patients with a mild form of MADD using indirect calorimetry and changes relevant in blood and urinary parameters during low-intensity prolonged exercise.

## Methods

We diagnosed two brothers with a milder form of MADD. The younger patient presented himself after an intercurrent illness at the age of 3.1 yr with a coma, hypoglycemia and metabolites, indicative for MADD. Assessment of his family revealed that the older brother was affected as well and in both boys the diagnosis was confirmed in cultured skin fibroblasts (ETF assay 0.30 and 0.22 nmol·min^-1^·mg^-1^protein for the older and younger brother respectively, controls 1.08 ± 0.16, Dr Vianey-Saban, Lyon). Both patients had regular carnitine suppletion (50–100 mg·kg^-1^·day^-1^) since the diagnosis.

The patients reported to the exercise laboratory after a light breakfast, and performed a graded maximal exercise test on the bicycle ergometer to determine the exercise intensity for the prolonged exercise test. The workload was increased in constant increments of 10 watts every 1 minute. This protocol continued until the patient stopped due to volitional exhaustion, despite strong verbal encouragement. During the test the subjects breathed through a facemask (Hans Rudolph Inc., Kansas City, MO) connected to a calibrated respiratory gas analysis system (Oxycon Champion, Jaeger, Bilthoven, The Netherlands) which measured and or calculated breath-by-breath minute ventilation (VE), oxygen uptake (VO_2_), carbon dioxide production (VCO_2_), and respiratory exchange ratio (RER; = VCO_2_·VO_2_^-1^) using conventional equations. Heart rate (HR) was measured continuously during the maximal exercise test by a bipolar electrocardiogram. Peak oxygen consumption (VO_2peak_) was taken as the average value over the last 30 seconds during the maximal exercise test. Efforts were considered to be at a maximum level if HR_peak _was > 180 per minute and RER_peak _was > 1.0 [7].

Urine samples were taken before and after (3 hours) the exercise test and analyzed for organic acid analysis. Blood samples were taken before and after exercise and analyzed for glucose, lactate, free fatty acids (FFA), ketone bodies, creatine kinase (CK), organic acids, and acyl-carnitines. In the afternoon, after complete recovery, isometric muscle strength from the quadriceps muscle was measured using a hand-held myometer (Penny and Giles, Christchurch, UK), as described by Bäckman [8], to exclude that the results obtained during the maximal exercise test were influenced by muscle weakness. The results of myometry were compared to current reference values [8].

The prolonged exercise test was performed on another visit to prevent interference of the previous test. This test consisted of 90 minutes cycling at a constant workload at 30 % of W_peak_. During this test the following variables were monitored: VO_2_, VCO_2, _HR and RER, using the same setting as reported above. For each period of gas collection, whole-body rates of carbohydrate and fat oxidation (g·min^-1^) were calculated from the rates of CO_2 _production and O_2 _uptake using the non-protein RER values, according to the following equations [9]:

CHO oxidation = 4.585 VCO_2 _(l·min^-1^) - 3.226 VO_2 _(l·min^-1^);

and

FAO = 1.695 VO_2 _(l·min^-1^) - 1.701 VCO_2 _(l·min^-1^);

These equations are based on the assumption that VO_2 _and VCO_2 _accurately reflect tissue O_2 _consumption and CO_2 _production. The energy provided from CHO and fat oxidation was calculated from their energy potentials (3.87 and 9.75 kcal·g^-1^, respectively) [9].

Blood samples were taken at regular time intervals (t = 0, 30, 60, 75 and 90 minutes after the start of exercise). Glucose, lactate, CK, ketone bodies, FFA, organic acids, and acyl-carnitines were tested and measured. Urine samples were taken 3 hours after exercise and analyzed for organic acids, carnitine and myoglobin.

Organic acid concentrations in urine and plasma were determined by gas chromatography-mass spectrometry as their trimethylsilyl derivatives (Hewlett Packard 5890 series II gas chromatograph linked to a HP 5989B MS-Engine mass spectrometer (Hewlett Packard, Avondale, PA). The coefficients of variation for the various measured organic acids varied between 10–15%. Analysis of acylcarnitines in plasma as their butyl esters was performed by electrospray tandem mass spectrometry, (ESI-MS-MS; Micromass Quattro Ultima, Micromass Ltd., UK) equipped with an Alliance HPLC system (Waters, Milford, MA, USA). Also for these analyses the coefficients of variation for the determined acylcarnitines were 10–15%.

The results of the exercise tests in these two boys with MADD were compared to the results of the same test protocol used in 5 boys with complaints of fatigue and exercise intolerance, in whom inborn errors of metabolism were excluded. Their characteristics can be appreciated from Table 1.

**Table 1:** Subject characteristics, exercise capacity and muscle strength

	Subject 1	Subject 2	Control subjects Mean (SD)
Age (years)	10.2	8.7	12.6 (2.6)
Weight (kg)	33 (SD-score 0)	25 (SD-score -1)	42.6 (6.5) (SD-score 0.01)
Height (cm)	143 (SD-score 0)	133 (SD-score 0)	154.6 (10.6) (SD-score -0.4)
W_peak _(Watt)	115	70	145 (70)
HR_peak _(b·min^-1^)	195	195	185 (5)
RER_peak_	1.27	1.25	1.22 (0.11)
VO_2peak _(L·min^-1^)	1.4 (SD-score -1.5)	1.2 (SD-score -1)	1.5 (0.46) (SD-score -1.6)
VO_2peak _(ml·kg^-1^·min^-1^)	42.8 (SD-score -1.65)	50.2 (SD-score 0.06)	35.3 (5.3) (SD-score -2.17)
Peak blood lactate 3 min after the exercise test (mmol·l^-1^)	7.3	6.0	6.5 (3.6)
Quadriceps muscle strength (N)	207 (SD-score 0.2)	156 (SD-score 0.8)	NM

*Abbreviations: *W_peak_: peak workload, HR_peak_: peak heart rate, RER_peak_: peak Respiratory Exchange Ratio, VO_2peak_: peak oxygen uptake, SD-score: standard deviation score, NM: not measured.

Informed consent was obtained from the parents after being fully informed about the test procedures and the possible risks involved. All procedures were approved by the Medical Ethics Committee of the University Medical Center Utrecht.

## Results

The results of the maximal exercise test and myometry are shown in Table 1. Both children had normal quadriceps muscle strength. Also, both children met the criteria for a maximal exercise test, i.e. a HR > 180·min^-1^, RER > 1.0 and increased blood lactate concentrations. Before and after the maximal exercise test none of the MADD specific metabolites were found in organic acid analysis and plasma acylcarnitines in subject 1 (16 mmol·mol^-1 ^creatinine in subject 1, normal value < 20), with in subject 2 only a marginal elevated urinary excretion of ethylmalonic acid (30 mmol·mol^-1 ^creatinine) in combination with normal plasma acylcarnitines.

**Figure 1:**
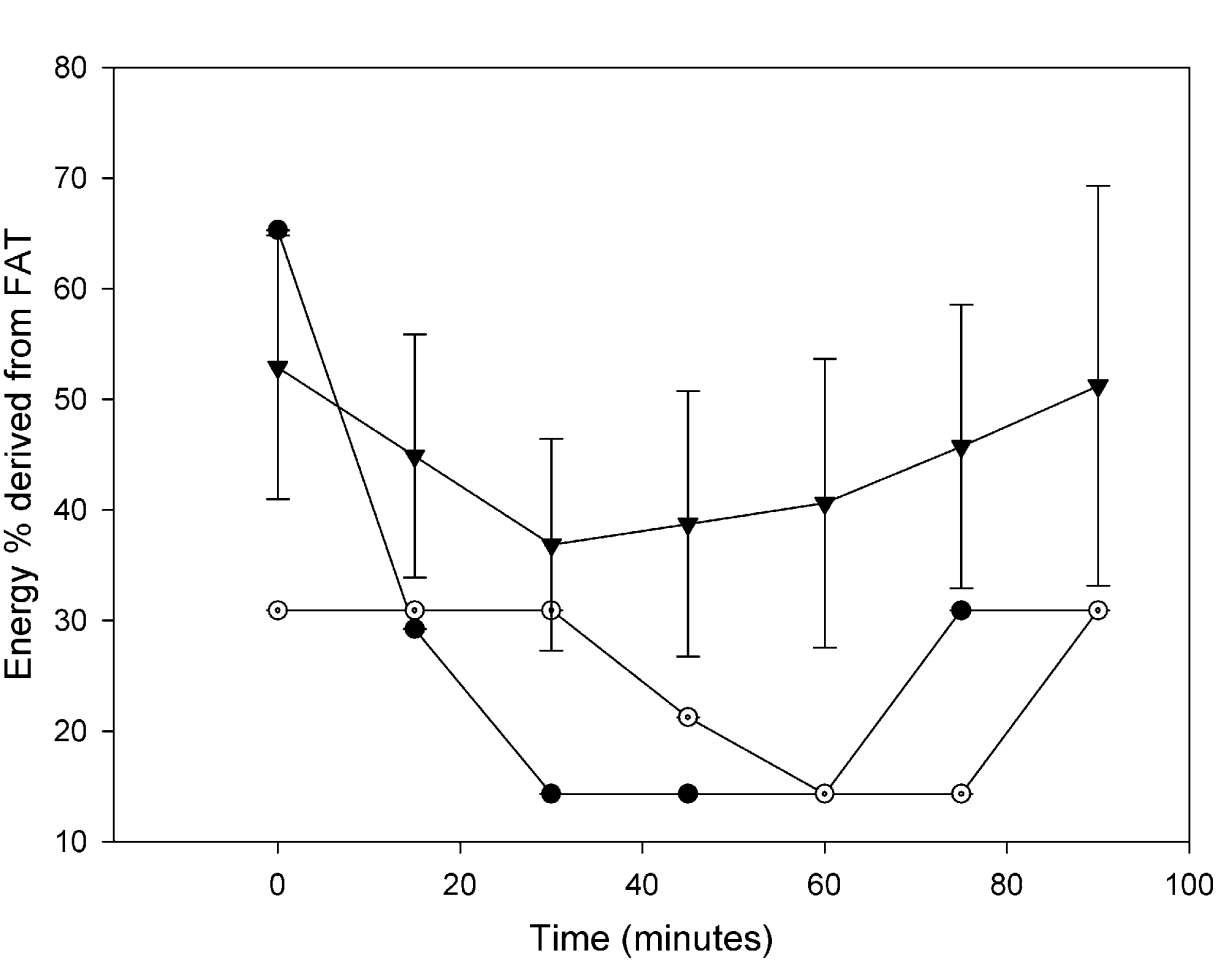
Percentage energy derived from fatty acid oxidation during the prolonged exercise test for patient 1 (filled circle), patient 2 (open circle) and 5 control subjects (black triangles). Values are mean ± SD.

Results of the prolonged exercise tests are shown in Figure 1 and Tables 2A and 2B. All patients did not reach the ventilatory anaerobic threshold during the 90-minutes exercise test, and had a VO_2 _of approximately 50% of VO_2peak_. All patients were very exhausted at the end of the prolonged exercise test. As can be seen in Figure 1 the FAO was very low in the two patients with MADD compared to the control subjects, although the exercise intensity was quite low (<50% VO_2peak_). Especially from 40 minutes onwards the difference in FAO was significant.

**Table 2A:** Results prolonged exercise test subject 1

	T = 0	T = 30	T = 60	T = 75	T = 90	T = 120
HR (b·min^-1^)	70	140	145	145	150	
VO_2 _(ml·min^-1^)	300	700	700	720	720	
RER (VCO_2_·VO_2_^-1^)	0.8	0.95	0.95	0.9	0.9	
Glucose (mmol·l^-1^)	7.1	5.6	4.9	4.8	5.5	4.8
Lactate (mmol·l^-1^)	1.1	1.2	1.0	1.3	1.1	1.0
CK (u·l^-1^)	103		98	105	119	117
3-keto-B (mmol·l^-1^)	0.07	<0.05	<0.05	0.05	0.1	0.15
3-OH-B (mmol·l^-1^)	0.09	<0.02	<0.02	0.05	0.12	0.26
FFA (umol·l^-1^)	0.76	0.28	0.49	0.88	1.03	0.93
**Urine**						
Ethylmalonic acid in Mmol·mol^-1 ^creatine	T = -60–0: 16					T = 120–300: 74

*Abbreviations: *HR: heart rate, RER: Respiratory Exchange Ratio, VO_2_: oxygen uptake, CK: creatine kinase, 3-keto-B: 3-ketobutyric acid, 3-OH-B: 3-hydroxybutyric acid, FFA: Free Fatty Acids.

**Table 2B:** Results prolonged exercise test subject 2.

	T = 0	T = 30	T = 60	T = 75	T = 90	T = 120
HR (b·min^-1^)	80	120	120	120	120	
VO_2 _(ml·min^-1^)	260	450	620	620	600	
RER (VCO_2_·VO_2_^-1^)	0.9	0.9	0.95	0.95	0.9	
Glucose (mmol·l^-1^)	5.2	4.6	4.3	4.4		4.5
Lactate (mmol·l^-1^)	2.0	0.9	0.9	1.0		1.2
CK (u·l^-1^)	58		80	78		78
3-keto-B (mmol·l^-1^)	<0.05	<0.05	0.07			0.14
3-OH-B (mmol·l^-1^)	<0.02	<0.02	0.04			0.24
FFA (umol·l^-1^)	0.15	0.31	0.62	0.91		1.34
**Urine**						
Ethylmalonic acid in mmol·mol^-1 ^creatinine	T = -60–0: 29					T = 120–300: 59

*Abbreviations: *HR: heart rate, RER: Respiratory Exchange Ratio, VO_2_: oxygen uptake, CK: creatine kinase, 3-keto-B: 3-ketobutyric acid, 3-OH-B: 3-hydroxybutyric acid, FFA: Free Fatty Acids.

Glucose, lactate and myoglobin levels did not change significantly during the tests. In both children levels of CK raised but stayed within normal limits. Under normal (resting) conditions plasma of both patients did not show any abnormality. However, ethylmalonic acid excretion rose to 74 mmol·mol^-1 ^creatinine in subject 1 and 59 mmol·mol^-1 ^creatinine in subject 2 and acylcarnitines showed characteristic short and medium chain acylcarnitines (details given later).

The urine and plasma of subject 1 and 2, collected after the prolonged exercise test, showed high excretions of metabolites diagnostic of MADD. In detail our analyses showed the following: in addition to ethylmalonic acid discussed earlier abnormal urinary compounds were found consisting of octanoic acid, 2-methyl-3-hydroxy-butyric acid, isobutyrylglycine, isovalerylglycine, 2-methylbutyrylglycine and hexanoylglycine (see tables 2A and 2B). After the prolonged exercise test (at T = 120 to 300), abnormal carnitine-ester profiles were seen in plasma samples of both patients. Elevations were noted of short and medium chain acylcarnitine esters (C-4-, C-6-, C-8-, C-10-, C-10:1-, C-12- and C-14:1-carnitine). Also free cis-decenoic acid was increased in the plasma samples after the prolonged exercise test (32 umol·L^-1 ^for subject 1 and 27 umol·L^-1 ^for subject 2, normal not detectable). Twenty-four hours after the exercise test, blood was drawn and analyzed for CK values and which showed normal results not different from baseline values.

After exercise the increased presence of ethylmalonic acid in urine with dicarboxilic acids combined with the abnormal short and medium chain acylcarnitine esters in plasma is diagnostic of dysfunctioning of multiple acyl-CoA dehydrogenases and therefore MADD. In the urine and plasma of the control subjects, no abnormal metabolites were found in organic acid analyses and acylcarnitines.

## Discussion

Little data are available on the risks and benefits of exercise in children with defects in cellular metabolism. It is well known that in milder forms of MADD, myopathy can be a prominent feature and there are no clear guidelines as to whether physical exercise should be avoided or advocated. Recent reports suggest that physical exercise might be beneficial for some patients with defects in energy metabolism [10,11]. It needs to be stressed that before exercise tests are being performed, a careful clinical evaluation is needed, including a complete cardiac evaluation, as cardiomyopathy is frequently present in patients with MADD. To evaluate the risks and advantages of exercise and training, exercise protocols need to be developed for different age groups and disorders. We think that an exercise protocol such as our protocol can be used to evaluate physical fitness and exercise risks in individual patients with a metabolic disease. However, optimal time and intensity of the prolonged exercise test remains to be determined.

The exercise tests that are currently used in our clinical setting (e.g. ischemic fore arm test and maximal exercise test) are not designed and therefore not appropriate for testing endurance exercise capacity. The same holds true for maximal exercise tests. Patients with MADD, even when their myopathy is mild, will probably not experience problems during the first 8–12 minutes of exercise. After maximal exercise, biochemical test results in patient 1 were not informative. In patient 2, before the maximal exercise test ethylmalonic acid was elevated 30 mmol·mol^-1 ^creatinine and did not rise during the test. Etylmalonic acid was the only abnormal metabolite present in the urine sample.

The recently used prolonged low-intensity exercise tests are of more value for evaluating patients with FAO disorders [5,6]. A prolonged exercise test designed for fatty acid oxidation disorders should be of low intensity < 70% of VO_2peak_, since below < 70% the fat oxidation is maximal [12]. We choose a duration of 90 minutes since this period was also used in previous exercise studies in patients with metabolic muscle diseases [13]. Recently others have used 60 minutes of cycling at 50% of VO_2peak _in patients with FAO disorders [6]. We have chosen to set the constant workload as a % of maximal workload instead of oxygen uptake, since oxygen uptake drifts during exercise [14]. From Figure 1 can be derived that the length should be at least 45 minutes to show significant differences between patients and controls. However, the greatest differences between the two patients and controls in FAO was at 60 minutes of exercise. Optimal time and intensity of the prolonged exercise test remains to be determined.

One would expect problems during prolonged endurance exercise. During low intensity exercise, the dominant substrates for the muscular activity are plasma free fatty acids (FFA) and muscle triglyceride, plasma glucose and muscle glycogen have only a minor role in the energy provision [15], this role is even lower in children [16]. As relative exercise intensity increases, there is a decrease in the proportion of the energy requirement derived from fat oxidation and an increase in that provided by carbohydrate oxidation [15]. During prolonged low-intensity exercise that can be maintained for 90 minutes or longer, there is a progressive decline in the proportion of energy derived from muscle glycogen and muscle triglyceride, while the oxidation of plasma free fatty acids will increase [15]. As to be expected, a maximal graded exercise test showed no abnormalities in our patients. However, when the prolonged exercise test was performed the characteristic metabolites of MADD appeared in urine and plasma (increased presence of ethylmalonic acid in urine combined with the abnormal mid-chain carnitine ester profile in plasma), without evident clinical or biochemical signs of muscle damage. Moreover, we found a low FAO during the low intensity prolonged exercise test as measured from indirect calorimetry in the two subjects with MADD compared to the 5 control subjects. The FAO oxidation was also lower compared to a previous study in healthy children [16], although in that study, the subjects even exercised at a higher exercise intensity (70% of VO_2peak_). Moreover, our data indicate that the low FAO is caused by an impaired FAO, and not caused by an impaired mobilization of plasma FFA, since plasma FFA levels were increasing during prolonged exercise.

Our results are comparable with the study of Orngreen et al [6] who tested 2 subject with very long-chain acyl-coa dehydrogenase deficiency (VLCAD) using a 60 endurance exercise test. In both patients with MADD and VLCAD subjects FAO was very low during exercise, and plasma glucose levels were decreasing during exercise, while plasma FFA concentrations were increasing during exercise.

When FAO disorders or MADD are suspected in a patient and the investigations of biological fluids does not reveal significant clues to the diagnosis, which sometimes is the case, "stress-tests" like a fasting test can be used. A fasting test can last up to 24 hours and is a less controlled tests compared to a standardized exercise test. Moreover, a fasting test is much more a burden to the patient than exercise tests. Furthermore, fasting tests do not give any guidance to clinicians in advising physical exercise possibilities or limitations in their patients. Given the fact that in the patients discussed in the manuscript the diagnosis MADD could be made after exercise suggests the diagnostic value of prolonged exercise testing.

## Conclusion

We performed a prolonged low-intensity exercise test in two boys with a mild form of MADD and 5 control subjects. The test was tolerated well in the 2 patients and the 5 control subjects, although they were exhausted at the end of the test. During the test the FAO was very low in the MADD compared to the control subjects, while specific metabolites of MADD appeared in plasma and urine samples. We suggest that this test could be of diagnostic importance and might replace the fasting test as a diagnostic procedure in some cases, particularly in patients with anamnestic signs of intolerance for prolonged exercise. Furthermore, based on the moment and rate of appearing of specific metabolites, a tailored counseling regarding physical activity can be given.

## Competing interests

The author(s) declare that they have no competing interests

## Authors' contributions

TT and JWHC carried out the exercise test and drafted the manuscript. TK, GV, LD carried out the analysis of blood and urine. TT, JWHC, TK, GV, LD, and PJMH conceived of the study, and participated in its design and coordination and helped to draft the manuscript. All authors read and approved the final manuscript.
